# *Helicobacter hepaticus* polysaccharide induces an anti-inflammatory response in intestinal macrophages

**DOI:** 10.15698/mic2018.04.626

**Published:** 2018-03-22

**Authors:** Camille Danne, Fiona Powrie

**Affiliations:** 1 Kennedy Institute of Rheumatology, University of Oxford, Oxford, UK.

**Keywords:** Helicobacter hepaticus, inflammatory bowel disease, host-microbiota interactions, mutualism, anti-inflammatory gene signature, polysaccharide, TLR2, CREB, MSK1/2, macrophage

## Abstract

A high density of microbes inhabits the intestine, helping with food digestion, vitamin synthesis, xenobiotic detoxification, pathogen resistance and immune system maturation. Crucial for human health, communities of commensal bacteria (collectively termed microbiota) benefit in return from a nutrient-rich environment. Host-microbiota mutualism results from a long-term co-adaptation. At barrier surfaces, immune cells distinguish harmful from commensal bacteria and tolerate non-self organisms at close proximity to the mucosa; gut inhabitants have developed strategies to ensure beneficial conditions in their preferred niche. So far, the complex dialogue of host-microbial mutualism is poorly understood. *Helicobacter hepaticus* is a member of the mouse microbiota that colonizes the lower intestine without inducing immune pathology. However, when there is a host maladaptation such as the absence of the anti-inflammatory cytokine interleukin 10 (IL-10) or its receptor IL-10R, *H. hepaticus* triggers aberrant IL-23-driven intestinal inflammation. This response results in major changes in the intestinal innate cell compartment, with the accumulation of inflammatory macrophages. Relying both on a bacterial trigger and on an immune defect, *H. hepaticus*-induced colitis in the context of IL-10/IL-10R axis deficiency shares many features of human inflammatory bowel diseases (IBD). In our study [Danne *et al*, Cell Host Microbe 22(6):733-745], we questioned the interactions between *H. hepaticus* and intestinal macrophages that promote mutualism. Our results show that *H. hepaticus* produces a large polysaccharide that triggers IL-10 production without a corresponding inflammatory response in macrophages. Moreover, *H. hepaticus* polysaccharide specifically induces an anti-inflammatory gene signature *in vitro* and *in vivo*, including transcriptional factors known as repressors of immune activation. This anti-inflammatory program depends on the TLR2/MSK/CREB pathway, which might be crucial to maintain mutualistic relationships at the intestinal interface.

 Strategically located at the mucosal barrier, intestinal macrophages are immune sentinels. Essential to homeostasis, they are sensors and interpreters of the intestinal microenvironment. They recognize microbial products from both commensals and pathogens through pattern-recognition receptors (PRRs), including Toll-like receptors (TLRs). The activation of PRRs triggers the production of pro-inflammatory mediators, such as IL-6 and TNFα; but also negative feedback mechanisms and anti-inflammatory signals, such as IL-10, to prevent chronic inflammation and tissue damage. However, the signaling mechanisms employed by particular gut inhabitants to actively promote mutualism remain elusive, as is their interaction with macrophages.

As blockade of IL-10 signaling induces colitis in *H. hepaticus-*colonized mice, we first assessed whether colonization of Specific Pathogen Free (SPF) mice with *H. hepaticus* triggers IL-10 production by intestinal macrophages. Three days post-inoculation, the frequency of IL10^high^ resident macrophages (MHCII^+^CD11b^+^CD11c^int/high ^CD64^+^) was increased in the caecal lamina propria. To examine the signaling mechanisms underlying IL-10 induction, we stimulated M-CSF-differentiated bone-marrow-derived macrophages (BMDMs) and showed that *H. hepaticus* culture supernatant (SN*Hh*) induces high IL-10 but low IL-6 and TNFα production compared to the whole bacteria, leading to high ratios of anti-inflammatory to inflammatory cytokines. IL-10 induction was not affected by treatment with DNase, RNase, and proteinase K followed by heat, suggesting that the active molecule in the resultant preparation (SN*Hh*t) was not a nucleic acid or a protein, but possibly a polysaccharide. Size fractionation showed that the ability to induce IL-10 in BMDMs was restricted to high molecular weight components, pointing towards a large polysaccharide. This conclusion was supported by the findings that treatment of SN*Hh*t with sodium metaperiodate, which cleaves polysaccharide chains, resulted in the loss of IL-10-inducing activity; and that the crude polysaccharide fraction isolated by cold ethanol precipitation recapitulated SN*Hh*t activity. Moreover, pre-incubation of SN*Hh*t with beads coated with the carbohydrate-binding lectin Concanavalin A depleted the IL-10-inducing activity, suggesting that the active molecule contains α-mannose and α-glucose sugars.

*In vivo*, oral administration of SN*Hh*t increased the frequency of IL-10-producing macrophages in the caecum of SPF mice. Intraperitoneal injection of SN*Hh*t induced *Il10 *expression in peritoneal cells, with a lower inflammatory response compared to canonical TLR ligands such as LPS from *Escherichia coli*, Pam2CSK4 or Pam3CSK4.

Experiments using BMDMs from a panel of PRR-deficient mice and pharmacological blockade of downstream kinases subsequently revealed that SN*Hh*t triggers IL-10 production through a mechanism involving TLR2, MyD88 and ERK1/2. Unlike other TLR2 agonists that elicit a pro-inflammatory response with high IL-6 and TNFα, *H. hepaticus* polysaccharide preferentially induces IL-10. To assess whole genome differences in the macrophage transcriptional response to SN*Hh*t or to the canonical TLR2/1 agonist Pam3CSK4, we performed a microarray analysis of BMDMs after 3h stimulation. It revealed that Pam3CSK4 induces a large repertoire of pro-inflammatory genes associated with M1 pro-inflammatory macrophages (*Il6*, *Saa3*, *Ccl5*) or involved in the recruitment, activation, and proliferation of T cells (*Cd40*, *Tnfrsf9*, *Icam1*); whereas SN*Hh*t specifically induces the transcription of genes highly expressed in tissue-resident or M2 macrophages (*Ccl7*, *Mmp13*, *Ptpn22*), transcription factors repressing NF-κB (*Rcan1*, *Atf3*) and T cell activation (*Egr3*), and genes involved in tissue repair (*Edn1*, *Mmp13*, *Hbegf*). Specific targets induced by SN*Hh*t were enriched for genes (*Rcan1*, *Egr3*, *Fosb)* with predicted binding sites for the anti-inflammatory transcription factor CREB. Using mice with targeted disruption of the key CREB phosphorylation site (conditional CREB^S133A KI^), or mice with complete knock-out of MSK1/2 kinases (*Msk1/2*^-/-^) -activated by ERK1/2 and responsible for CREB phosphorylation-, we confirmed that both MSK1/2 and CREB are required for SN*Hh*t anti-inflammatory properties. SN*Hh*t also induces a CREB/MSK-dependent immunomodulatory program in intestinal tissue *in vivo*, raising the possibility that it contributes to mutualistic relationships in the *H. hepaticus-*infected gut.

This work raises questions about the structure of *H. hepaticus* polysaccharide, the genes and conditions required for its production and the strain and species specificity of its activity (discussed in the original publication). Considering the high molecular weight and presence in the culture supernatant,* H. hepaticus* polysaccharide could be carried by Outer Membrane Vesicles (OMVs), as described for the anti-inflammatory Polysaccharide A (PSA) from *Bacteroides fragilis*. Electron microscopy revealed OMVs in SN*Hh* that were not present in SN*Hh*t, probably due to the high temperature treatment. Analysis of purified OMVs is necessary to determine if the polysaccharide is a component of these structures.

In the *H. hepaticus* +αIL-10R model, TLR2^-/-^ mice did not develop stronger intestinal inflammation compared to wild-type mice, possibly because the severity is already high in wild-type conditions with neutralization of IL-10. However, it is difficult to draw conclusions from this experiment as TLR2 mediates both pro- and anti-inflammatory responses. Moreover, SN*Hh*t strongly activates CREB compared to the TLR2 agonist Pam3CSK4, which suggests that SN*Hh*t signaling may result from the interaction of TLR2 with another receptor, even though we have ruled out several candidates such as C-type lectins (CLRs) and the downstream CLR-signaling protein CARD9. Further studies are required to determine the contribution of the *H. hepaticus* polysaccharide-driven TLR2/CREB-dependent response in macrophages to *H. hepaticus* infection *in vivo*; and address whether the CREB/MSK pathway controls intestinal inflammation in colitis models.

Drawing the line between commensals and pathogens is difficult, but health-promoting properties of certain gut inhabitants are well-documented. By degrading indigestible fibers, anaerobic species produce Short Chain Fatty Acids (SCFAs), which exert multiple beneficial effects on cellular immunity and metabolism. Bifidobacteria are primary degraders of diet- and host-derived sugars, providing SCFAs for the host and nutrients for other community members. They also interact directly with immune cells and modulate both innate and adaptive immune processes, such as IL-10 production and T cell induction. Several immunomodulatory molecules have been identified in Bifidobacteria, including the extracellular serpin that inactivates human pro-inflammatory proteases and anti-inflammatory exopolysaccharides*. *Another predominant species of the indigenous microbiota, *Faecalibacterium prausnitzii* from the *Clostridium* cluster IV, is directly associated with intestinal health and reduced in several diseases including IBD.* F. prausnitzii *is a strong producer of the SCFA butyrate and releases in its supernatant the protein MAM (Microbial Anti-inflammatory Molecule), which prevents intestinal inflammation by inhibiting NF-κb and the production of Th1/Th17 cytokines. Although Lactobacillus is a minor member of the human colonic microbiota, intestinal depletion of certain species is associated with chronic diseases. Recently, the capacity of Lactobacillus strains to metabolize tryptophan into AhR ligands was shown to attenuate intestinal inflammation in mice. Sometimes, two strains from the same species have distinct effects on host physiology depending on the expression of one virulence or tolerogenic factor. For example, *B. fragilis* can be highly pathogenic in mice and humans due to the production of a toxin, whereas the PSA-expressing strain induces an immunomodulatory response in murine models.

Between the extremes of "friend" and "foe" categories, a variety of opportunistic pathogens and pathobionts thrive in the intestinal niche by escaping immune defenses. The microbes residing in the upper mucus layer stay at a safe distance from the epithelium, but the persistent colonizers living in close contact with the mucosa have to encourage immune tolerance to avoid chronic activation and deleterious tissue damage. As described above, *H. hepaticus* releases a polysaccharide inducing a specific CREB-dependent anti-inflammatory program in the intestine, which might be essential to tolerize the niche and maintain intestinal homeostasis. *Helicobacter pylori, *a frequent colonizer of the human gastric mucosa, can cause severe inflammation and gastric tumorigenesis, but also protects against esophageal cancer and chronic inflammatory disorders. It has evolved to skew the adaptive immune response toward immune tolerance rather than immunity, which promotes persistent infection but also protects against allergic and auto-immune diseases. For instance, due to structural modifications,* H. pylori* flagellin and LPS are poor activators of TLR5 and TLR4 receptors respectively; and its TLR2 ligands exhibit predominantly anti-inflammatory properties. The intimate contact with *H. pylori* generates a limited but constant inflammation that can become deleterious to the host, depending on strain-specific virulence determinants, host genetics, environment and surrounding microbial communities (Figure 1).

**Figure 1:**
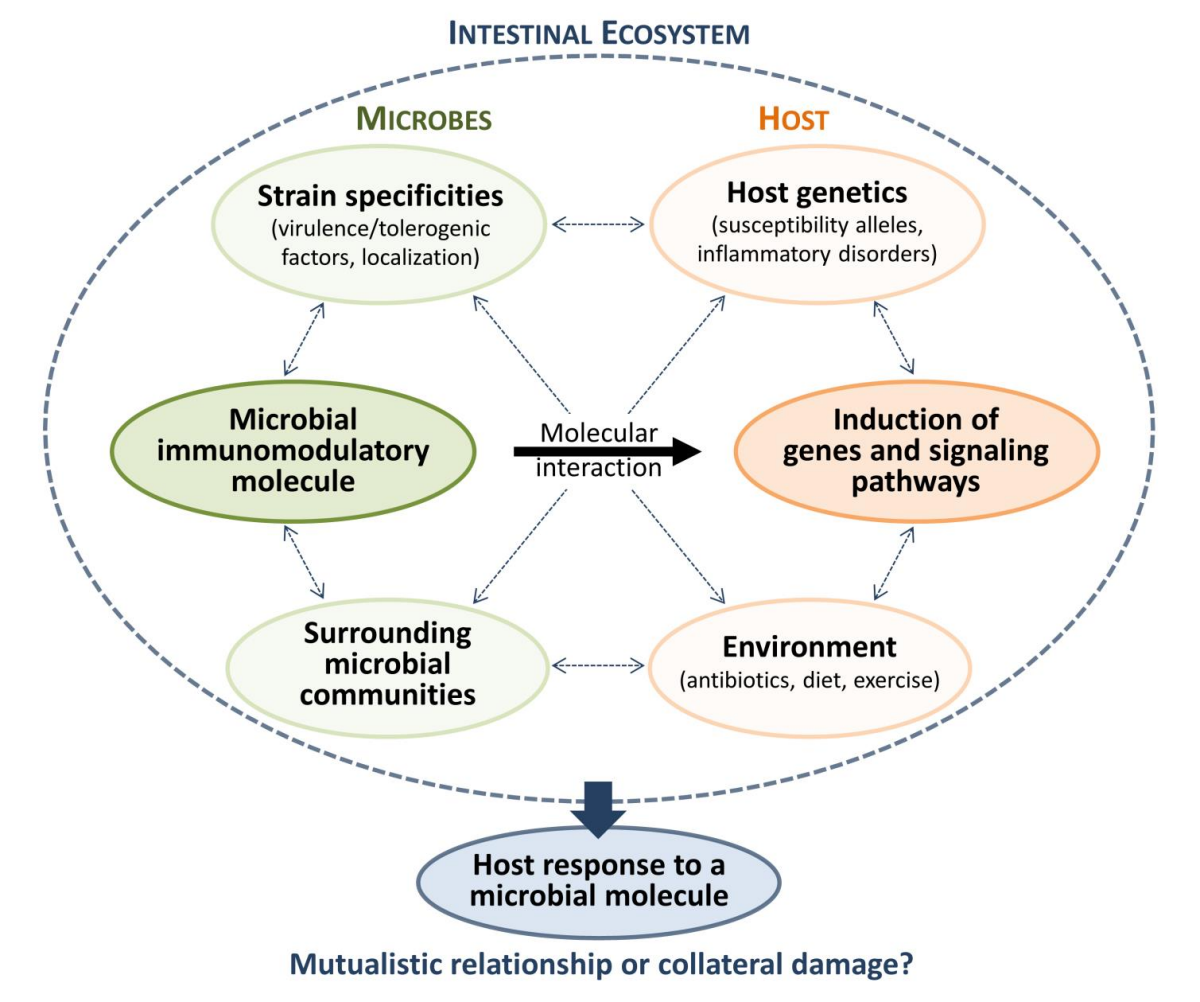
FIGURE 1: Flow chart representing host, microbe and environmental factors that influence the host response to a microbial immunomodulatory molecule. These complex interactions determine whether a specific host-microbe interaction contributes to mutualism or leads to collateral damage in the long term.

The intestinal microbiota contains an inherent capacity to trigger immunomodulatory responses that are essential to maintain health. Characterising the diversity of factors that promote mutualism and their modes of action at the cellular and molecular level is crucial to elucidate host-microbiota crosstalk. Modulation of immunological processes in a microbe-dependent way offers the prospect of innovative strategies to treat and prevent chronic intestinal inflammation.

